# Anti-inflammatory and Cytotoxic Triterpenes from the Rot Roots of *Panax notoginseng*

**DOI:** 10.1007/s13659-019-0211-4

**Published:** 2019-05-23

**Authors:** Jia-Huan Shang, Guo-Wei Xu, Hong-Tao Zhu, Dong Wang, Chong-Ren Yang, Ying-Jun Zhang

**Affiliations:** 1 State Key Laboratory of Phytochemistry and Plant Resources in West China, Kunming Institute of Botany, Chinese Academy of Sciences, Kunming, 650201 People’s Republic of China; 2 University of Chinese Academy of Sciences, Beijing, 100049 People’s Republic of China; 3 Yunnan Key Laboratory of Natural Medicinal Chemistry, Kunming Institute of Botany, Chinese Academy of Sciences, Kunming, 650201 People’s Republic of China

**Keywords:** *Panax notoginseng*, Rot root, Triterpenes and saponins, Inhibition on NO production, Cytotoxicity

## Abstract

**Abstract:**

Four new protopanaxatriol-type triterpenes (**1**–**2**) and glucosides (**3**–**4**), were isolated from the rot roots of *Panax notoginseng* (Burk.) Chen, along with four known ones (**5**–**8**). Their structures were elucidated on the basis of extensive spectroscopic analysis (HRESIMS, NMR, UV, IR, and OR) and acidic hydrolysis. The possible transformation pathway of these compounds were also speculated from ginsenoside Rg_1_. Compound **1**, with a unique α,β-unsaturated ketene in its side chain, showed significant inhibitory effects against NO production on Murine macrophage cells (IC_50_ = 4.12 ± 0.20 μM) and comparable cytotoxicities against five human cancer cell lines (myeloid leukemia HL-60, lung cancer A-549 cells, hepatocellular carcinoma SMMC7721, breast cancer MCF-7, and colon cancer SW480) to positive control, cisplatin (DDP).

**Graphical Abstract:**

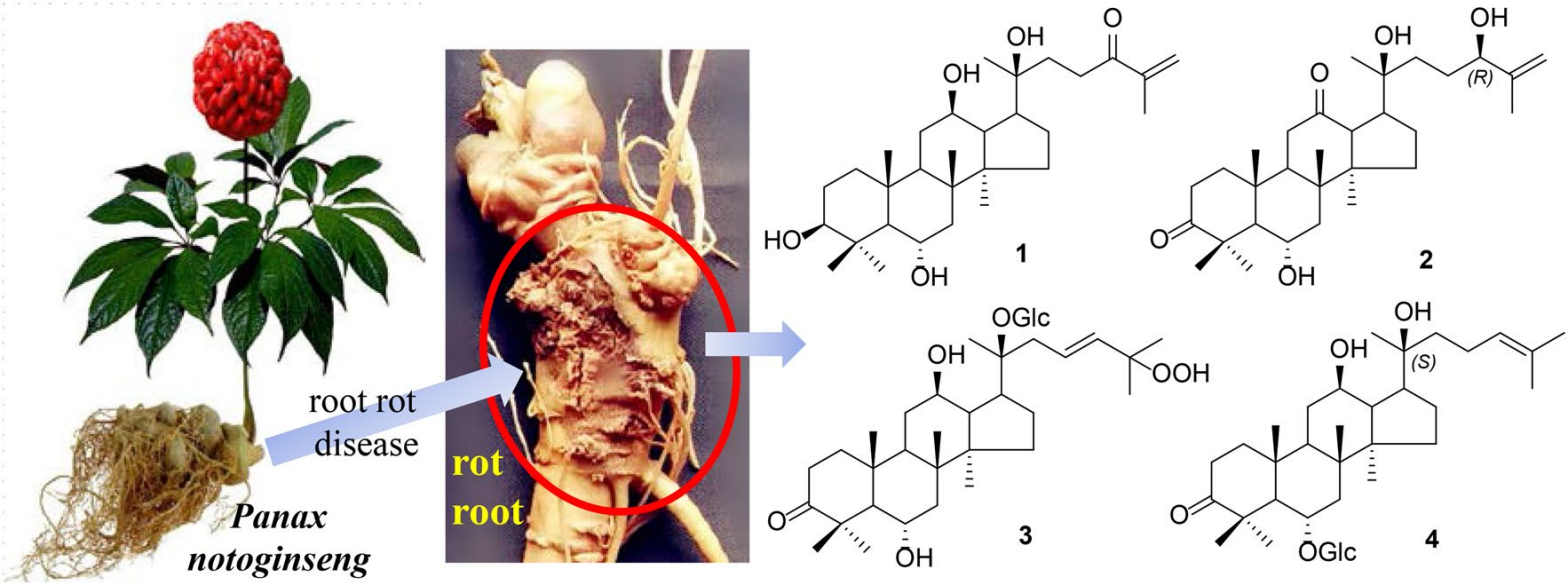

**Electronic supplementary material:**

The online version of this article (10.1007/s13659-019-0211-4) contains supplementary material, which is available to authorized users.

## Introduction

*Panax notoginseng* (Burk.) Chen (Araliaceae), a well-known member called Sanqi or Tianqi in Ginseng family for the treatment of cardiovascular diseases, has been domesticated and cultivated for more than 400 years in the southwest of China [1]. It is now used as one of the major raw materials for many traditional Chinese medicinal preparations, such as Pien Tze Huang, Yunnan Baiyao, Sanqi oral liquid, etc. [2]. Due to the increasing demands from medicinal industry and herbal market, the plantation of Sanqi has been enlarged sharply in recent decades. However, it is susceptible to various diseases during the plantation, because of the sensitive property to environmental factors. Among which, root rot caused mainly by microbial infections is the main destructive disease [3–5].

**Fig. 1:**
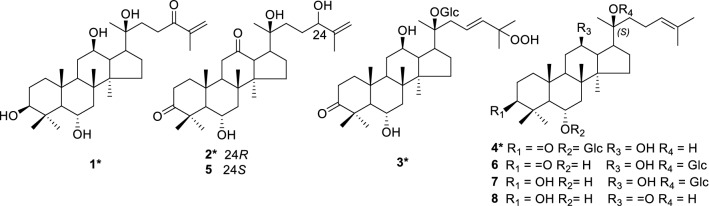
Compounds **1**–**8** isolated from the rot roots of *P. notoginseng*

So far, more than 200 chemical compounds including triterpenoid saponins, flavonoids, amino acids, and so on, have been reported from the roots, stems, leaves, flower heads, fruits, and fruit pedicels, and ginsenosides are found to be the dominant active principles [6]. Previous HPLC-HRMS study revealed that the oxidation levels of constituents in roots of *P. notoginseng* are significantly increased after being infected by root rot diseases [7]. In order to clarify the chemical composition and explore the possible transformation mechanism in the rot roots of *P. notoginseng*, the minor chemical constituents of the rot root were studied in detail firstly. This led to the identification of four new (**1**–**4**) and four known (**5**–**8**) triterpenes and saponins (Fig. 1), and their possible transformation pathway were speculated in this paper. All the isolates were evaluated for their anti-inflammatory activity (inhibition NO production) on Murine macrophage cells and cytotoxicities against five human cancer cell lines.

## Results and discussion

The air-dried rot roots of *P. notoginseng* were crushed into small grains and extracted with MeOH. Further repeated column chromatography (CC) over macroporous resin D101, silica gel and RP-18, followed by semi-preparative HPLC, yielded eight dammarane type triterpenes. Four of them (**1**–**4**) including two saponins (**3**–**4**) are new compounds.

**Fig. 2:**
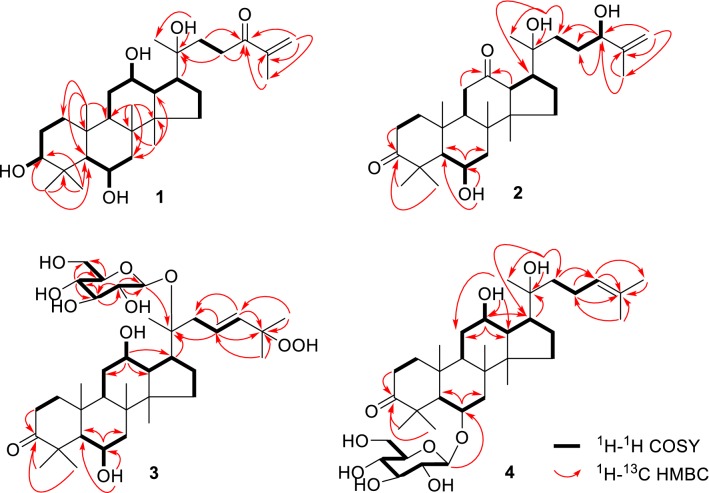
Key ^1^H-^1^H COSY and ^1^H-^13^C HMBC correlations of compounds **1**–**4**

Compound **1** was obtained as white amorphous powder. Its molecular formula C_30_H_50_O_5_ was determined by the positive-mode HRESIMS (*m/z* 513.3551 [M+Na]^+^), corresponding to six degrees of unsaturation. The IR spectrum indicated the presence of hydroxy groups (3391 cm^−1^), keto (1738 cm^−1^), double bond (1674 cm^−1^) and methyls (2954 cm^−1^). The UV spectrum presented a weak absorption at 254 nm (K-band) indicating the existence of a conjugated α,β-unsaturated ketene in this structure. The ^13^C NMR spectrum (Table 1) showed the presence of 30 carbon resonances, assignable to seven methyls, nine methylenes with a vinyl (*δ*_C_ 124.9), seven methines with three oxygen-bearing ones (*δ*_C_ 68.1, 71.6 and 78.9), and seven quaternary carbons, including one oxygenated carbon (*δ*_C_ 72.9), one olefinic carbon (*δ*_C_ 145.2) and one ketone (*δ*_C_ 203.2). The ^1^H NMR spectrum (Table 2) displayed seven singlet methyls at *δ*_H_ 2.02, 1.89, 1.47, 1.38, 1.10, 1.01 and 0.97 (each 3H, s), two olefinic protons at *δ*_H_ 6.06 and 5.66 (each 1H, s), three oxymethines at *δ*_H_ 4.43 (t, *J* = 8.5 Hz), 3.54 (d, *J* = 10.8 Hz), and 3.95 (t, *J* = 10.8 Hz), and four active hydrogen protons at *δ*_H_ 7.39, 7.02, 5.79, 5.32 (each 1H, s). The two of six unsaturated degrees were from the carbonyl and double bond. Above analysis displayed **1** to be a tetracyclic triterpene. The NMR data of **1** were similar to those of 20(*S*)-protopanaxatriol (PPT) [8], except for an additional ketone (*δ*_C_ 203.2), the shielding of C-25 (*δ*_C_ 145.2 vs *δ*_C_ 150.4 in PPT), and the appearance of a terminal vinyl at C-25 (*δ*_C_ 124.9). This indicated **1** to be a PPT-type triterpene with an α,β-unsaturated ketone (C-24) on the side chain. The ^1^H-^1^H COSY correlations from H_2_-22 (*δ*_H_ 3.31, 3.02) to H_2_-23 (*δ*_H_ 2.39, 2.05) combined with the HMBC spectrum of H-22/H-23/H_2_-26 (*δ*_H_ 6.06, 5.66)/H-27 (*δ*_H_ 1.89) to C-24 (*δ*_C_ 203.2), H-26/H-27 to C-25 (*δ*_C_ 145.2), H-17 (*δ*_H_ 2.37)/H-21 (*δ*_H_ 1.38)/H-22/H-23 to C-20 (*δ*_C_ 72.9), and of an active hydrogen signals (20-OH, *δ*_H_ 7.02) to C-20/C-21 (*δ*_C_ 27.5) (Fig. 2), respectively, further verified the structure of **1**. The 20*S* configuration in **1** was constructed by comparison of the chemical shifts of C-21 (*δ*_C_ 27.5) and C-17 (*δ*_C_ 55.2) with those of 20*R* configuration [C-21 (*δ*_C_ 22.8) and C-17 (*δ*_C_ 50.7)] [9, 10]. Therefore, compound **1** was identified as 3*β*,6*α*,12*β*,20(*S*)-tetrahydroxydammar-24-one-25-ene.

**Table 1:** ^13^C (150 MHz) NMR data of **1**–**4** in pyridine-*d*_5_ (*δ* in ppm)

No.	**1**	**2**	**3**	**4**	No.	**1**	**2**	**3**	**4**
1	39.8	39.7	40.3	40.5	19	17.9	17.9	17.5	18.7
2	28.6	33.5	33.8	33.7	20	72.9	73.7	83.5	73.5
3	78.9	218.7	219.1	219.0	21	27.5	27.4	23.7	27.5
4	40.8	48.1	48.1	48.6	22	33.2	38.9	40.3	36.3
5	62.2	59.1	59.5	58.4	23	30.5	31.4	127.0	23.5
6	68.1	67.2	67.3	79.8	24	203.2	76.5	138.6	126.8
7	48.0	45.1	45.8	43.8	25	145.2	150.4	81.8	131.3
8	41.6	41.6	41.0	40.7	26	124.9	110.8	25.6	26.3
9	50.6	53.5	49.0	48.8	27	18.3	18.4	25.9	18.2
10	39.8	38.6	38.6	38.9	28	32.4	32.4	32.6	32.3
11	32.6	40.6	32.2	33.3	29	17.0	20.3	20.5	20.2
12	71.6	211.4	70.7	71.2	30	17.5	17.4	16.7	17.1
13	48.6	56.5	49.8	49.3	1′			98.8	105.8
14	52.1	56.3	51.9	52.2	2′			75.8	75.9
15	31.8	32.6	31.1	31.7	3′			79.4	80.1
16	27.3	24.9	26.8	27.3	4′			72.1	72.4
17	55.2	44.4	52.5	55.1	5′			78.8	78.6
18	18.0	16.3	18.2	16.5	6′			63.5	63.6

**Table 2:** ^1^H (600 MHz) NMR data of **1**–**4** in pyridine-*d*_5_ (*δ* in ppm, *J* in Hz)

No.	**1**	**2**	**3**	**4**
1	1.68 m, 1.03 m	1.55 m	1.80 m, 1.59 m	1.75 m, 1.56 m
2	1.93 m, 1.86 m	2.81 m, 2.30 m	2.80 m, 2.32 m	2.83 m, 2.31 m
3	3.54 d (10.8)			
OH-3	5.79 s			
5	1.23 d (10.4)	1.95 d (10.8)	1.93 m	2.15 d (10.6)
6	4.43 t (8.5)	4.27 m	4.23 m	4.29 td (10.7, 3.8)
OH-6	5.32 s	5.85 s	5.71 s	
7	1.98 m	1.93 m	1.88 m	2.64 dd (12.9, 3.8)
	1.91 m	1.89 m		1.93 d (12.1)
9	1.60 m	1.99 dd (13.0, 4.3)	1.66 m	2.06 m
11	2.16 m, 1.25 m	2.31 m	2.03 m, 1.53 m	2.04 m, 2.15 m
12	3.95 t (10.8)		4.03 m	3.90 m
OH-12	7.39 s		7.40 s	7.29 s
13	2.09 t (10.9)	3.43 d (9.6)	2.01 m	1.66 m
15	1.58 m	1.88 m, 1.19 m	1.51 m, 1.01 m	1.69 m, 1.28 m
16	1.88 m, 1.42 m	2.16 m, 1.88 m	1.80 m, 1.44 m	1.84 m, 1.38 m
17	2.37 m	2.78 m	2.49 m	2.34 m
18	1.01 s	1.24 s	0.88 s	1.13 s
19	1.10 s	0.82 s	0.74 s	0.88 s
OH-20	7.02	5.48		7.03
21	1.38 s	1.47 s	1.62 s	1.43 s
22	3.31 m	2.15 m	3.08 dd (14.1, 6.1)	2.05 m
	3.02 m	1.88 m	2.76 m	2.71 m
23	2.39 m, 2.05 m	2.19 m, 2.02 m	6.21 m	2.62 m, 2.29 m
24		4.42 t (6.0)	6.08 d (15.7)	5.35 tt (7.1, 1.2)
OH-24		6.41 s		
OOH-25			14.37 s	
26	6.06 s, 5.66 s	5.25 m, 4.97 m	1.59 s	1.68 s
27	1.89 s	1.92 s	1.60 s	1.64 s
28	2.02 s	1.67 s	1.68 s	1.79 s
29	1.47 s	1.70 s	1.73 s	1.89 s
30	0.97 s	0.91 s	1.09 s	0.88 s
1′			5.26 d (7.7)	5.04 d (7.8)
2′			4.04 m	4.08 m
3′			4.27 m	4.25 m
4′			4.17 m	4.23 m
5′			4.03 m	3.96 m
6′			4.55 d (11.4)	4.57 d (11.3)
			4.35 m	4.38 m

Compound **2** presented a molecular formula C_30_H_48_O_5_ with seven degrees of unsaturation as deduced from HRESIMS experiment (*m/z* 511.3399 [M+Na]^+^). The ^13^C NMR spectrum of **2** showed an olefinic (*δ*_C_ 150.4, 110.8), two carbonyl groups (*δ*_C_ 218.7, 211.4), while the ^1^H NMR indicated the existence of three active hydrogens at *δ*_H_ 5.48, *δ*_H_ 5.87, *δ*_H_ 6.46, two oxymethines at *δ*_H_ 4.42 (t, *J* = 6.0 Hz), *δ*_H_ 4.27, and two vinyl protons at *δ*_H_ 5.25 and *δ*_H_ 4.97. An oxygen-bearing methine at *δ*_H_ 4.42 (H-24) was correlated with *δ*_C_ 31.4 (C-23)/*δ*_C_ 150.4 (C-25)/*δ*_C_ 18.4 (C-27) in HMBC spectrum (Fig. 2), suggesting a terminal enol moiety in the side chain. The ^1^H and ^13^C NMR data of **2** were strikingly similar to those of 6*α*,20(*S*),24(*S*)-trihydroxydammar-3,12-dione-25-ene (**5**) [11], which was obtained from semi-preparative HPLC together with **2**, showing discernible retention times (28.903 min for **5** and 31.587 min for **2**) (SI). Dammanrane-type triterpenes, deriving from the all-chair formed epoxysqualene, requires *β*-configuration of H-13, CH_3_-18, CH_3_-19 and the side chain at C-17 [12]. ROESY correlations from H-17 to H-13 and CH_3_-21 further facilitated assignment of the 20*S*-configuration for both **2** and **5** [13]. The very small difference for the upfield shift of H-26a (*δ*_H_ 5.25 vs *δ*_H_ 5.28 in **5**), C-27 (*δ*_C_ 18.4 vs *δ*_C_ 18.8 in **5**) and downfield shift of H-27 (*δ*_H_ 1.92 vs *δ*_H_ 1.90 in **5**), C24-C26 (*δ*_C_ 76.4, 150.4, 110.8 vs *δ*_C_ 76.3, 150.3, 110.6 in **5**) suggested **2** to be a stereoisomer of **5** at C-24. Compound **2** showed a positive optical rotation ($[\alpha]_{\text{D}}^{25}$ + 94.95), which is opposite to that of **5** ($[\alpha]_{\text{D}}^{25}$ − 51.5) [11], revealed the 24*R*-configuration in **2**. Accordingly, compound **2** was determined to be 6*α*,20(*S*),24(*R*)-trihydroxydammar-3,12-dione-25-ene.

Compound **3** was found to have a molecular formula of C_36_H_60_O_11_ on basis of the positive-mode HRESIMS experiment (*m/z* 691.4024 [M+Na]^+^). The ^13^C NMR and DEPT spectra showed six more carbon resonances than those of **1**, suggesting the existence of a hexosyl unit (*δ*_C_ 98.8 d, 79.4 d, 78.8 d, 75.8 d, 72.1 d, 63.5 t). So far, all hexosyl units in ginsenosides isolated from *Panax* species is only glucosyl (Glc) [14]. Further acidic hydrolysis of **3** followed by GC analysis of the corresponding trimethylsilylated l-cysteine adduct characterized a glucopyranosyl moiety in compound **3**. The ^1^H-NMR spectrum of **3** showed an anomeric proton signal of the *β*-d-glucopyranosyl moiety at *δ*_H_ 5.26 (d, *J *= 7.7 Hz), an active hydrogen at *δ*_H_ 14.37 (s) and two olefinic protons in a disubstituted *E*-double bond at *δ*_H_ 6.08 (d, *J *= 15.7 Hz) and *δ*_H_ 6.21 (m). The ^13^C and ^1^H NMR data of **3** were similar to those of a ginsenoside-Rh_6_ [15], except for the signals of C1–C5 at ring A. The very down field chemical shift at *δ*_C_ 219.1 suggested that **3** should be 3-oxidized ginsenoside-Rh_6_ as the hydroxy group at C-3 (*δ*_C_ 78.6) in ginsenoside-Rh_6_ was transferred to a ketone group. This was further verified by the HMBC correlations from H_2_-2 (*δ*_H_ 2.82, 2.32), H-28 (*δ*_H_ 1.68) and H-29 (*δ*_H_ 1.73) to C-3 (*δ*_C_ 219.1). Moreover, on the basic of the correlations of *δ*_H_ 14.37 (–OOH) with C-25 (*δ*_C_ 81.8), H-1′ (*δ*_H_ 5.26) with C-20 (*δ*_C_ 83.5) in HMBC spectrum and H-1′with H-21 (*δ*_H_ 1.62, s)/H_2_-22 (*δ*_H_ 3.08 dd, *J *= 14.1, 6.1 Hz; *δ*_H_ 2.76 m)/H-23 (*δ*_H_ 6.21, m) in ROESY spectrum, the positions of peroxy and glucosyl groups were confirmed to be linked with C-25 and C-20, respectively. Therefore, compound **3** was assigned as 3-oxo-20(*S*)-ginsenoside-Rh_6_.

Compound **4** was an amorphous white powder and had a molecular formula C_36_H_60_O_9_, as deduced from HRESIMS experiment (*m/z* 659.4126 [M+Na]^+^). An *β*-d-glucopyranosyl unit (*δ*_C_ 105.8 d, 80.1 d, 78.6 d, 75.9 d, 72.4 d, 63.6 t) could be recognized by ^13^C NMR data and the anomeric proton at *δ*_H_ 5.04 (d, *J *= 7.8 Hz), and confirmed by GC analysis after acidic hydrolysis. Furthermore, the HMBC correlations from H-5/H-7/H-1′ to C-6 (*δ*_C_ 79.8) indicated the glucosyl group was substituted at C-6. The ^1^H and ^13^C NMR data of **4** were in good agreement with those of 20(*S*)-ginsenoside-Rh_1_ [16], except for signals of ring A, which showed a lower field quaternary carbon at *δ*_C_ 219.0. Similar to those of **2** and **3**, H_2_-2 (*δ*_H_ 2.83, 2.31), CH_3_-28 (*δ*_H_ 1.79, s) and CH_3_-29 (*δ*_H_ 1.89, s) displayed HMBC correlations with *δ*_C_ 219.0, indicating that **4** was an oxidized ginsenoside-Rh_1_ at C-3. The absolute configuration of C-20 (*δ*_C_ 73.5) was identified as 20*S* when compared its surrounding carbon signals [C-17 (*δ*_C_ 55.1), C-21 (*δ*_C_ 27.5) and C-22 (*δ*_C_ 36.3)] with that in 3-oxo-20(*R*)-ginsenoside-Rh_1_ [C-17 (*δ*_C_ 50.9), C-21 (*δ*_C_ 23.2), and C-22 (*δ*_C_ 43.6)] [17]. Accordingly, compound **4** was identified as 3-oxo-20(*S*)-ginsenoside-Rh_1_.

**Fig. 3:**
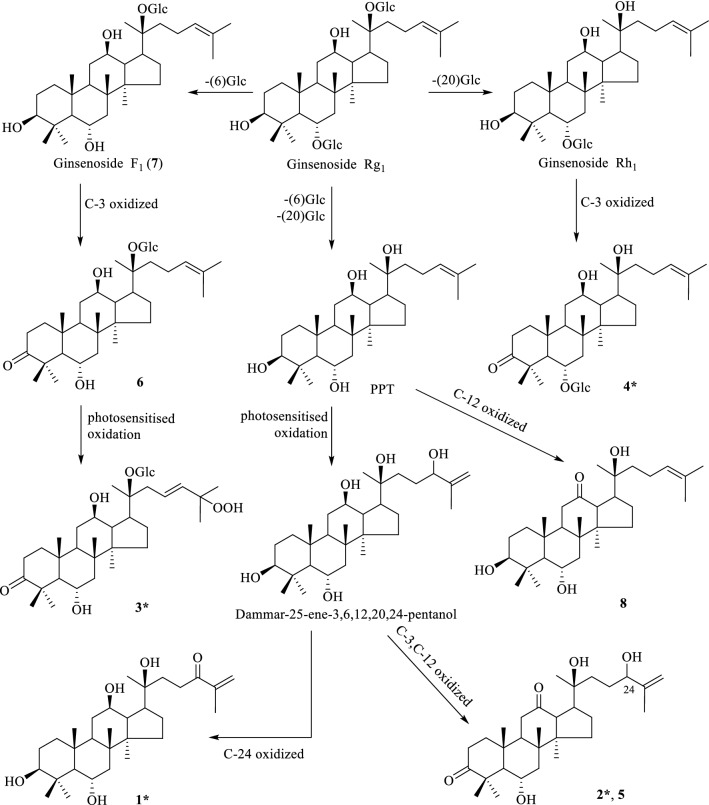
The possible transformation pathway of **1**–**8** from ginsenoside Rg_1_

Four known compounds were identified as 6*α*,20(*S*),24(*S*)-trihydroxydammar-3,12-dione-25-ene (**5**) [11], ginsenoside-F_1_ (**6**) [18], 12-oxo-20(*S*)-protopanaxatriol (**7**) [19], 6*α*,12*β*-dihydroxydammar-3-one-20(*S*)-*O*-*β*-d-glucopyranoside (8) [20], respectively, by comparison of their spectroscopic data with literature reported values. Related substrate specificity test probed that the glucoside bond of ginsenoside Rg_1_ can be selectively hydrolyzed by *β*-d-glucosidase and thus produced rare ginsenoside F_1_ [21]. Accordingly, *β*-d-glucosidase is widespread in fungi [22] and bacteria [23], and the oxidative stress response in this herb after infected by the root rot decease might play the main role for the oxidation of hydroxyl group at C-3, C-12, or the side chain. As all the eight compounds are PPT-type triterpenes, we speculated that they might be transformed from ginsenoside Rg_1_, the most abundant PPT-type saponin in the root of *P. notoginseng,* and the possible transformation pathway of these eight compounds were deduced as Fig. 3.

Compound **1** is a triterpene aglycone with a terminal α,β-unsaturated ketene on the side chain, whose diglycoside and triglycoside in flower buds of *P. notoginseng* and tetraglycoside in Xueshuantong Injection were reported previously by LC–MS(Q-TOF) analysis [24, 25]. In the present study, compound **1** was isolated and identified with other three new compounds (**2**–**4**) and four known ones from the rot roots of *P. notoginseng* for the first time. Compounds **2** and **5** were a pair of diastereoisomers and separated simultaneously by semi-preparative HPLC.

Since the glycosides in *Panax* species were reported to have strong anti-inflammatory and anti-tumor activities [24–30], all the isolates were evaluated for their inhibitory effects against NO production on Murine macrophage cell line and cytotoxicities against five human cancer cells (myeloid leukemia HL-60, lung cancer A-549 cells, hepatocellular carcinoma SMMC7721, breast cancer MCF-7, and colon cancer SW480). Only compound **1** displayed significant inhibition of NO production with IC_50_ value of 4.12 ± 0.2 μM (L-NMMA: IC_50_ = 34.32 ± 1.20 μM) and obvious cytoxicities against all the five human cancer cell lines, comparable to the positive control, DDP (Table 3). At primary concentration of 50 μM for NO inhibition and 40 μM for cytotoxicity, the other compounds **2**–**8** displayed no obvious anti-inflammatory and cytotoxic activities.

**Table 3:** Cytotoxicities of compound **1** against five human cancer cell lines

Cancer cells	IC_50_ ± SD (μM)		
	**1**	DDP	Taxol
Myeloid leukemia HL-60	19.12 ± 0.21	2.87 ± 0.08	< 0.008
Lung cancer A-549	16.30 ± 0.21	20.62 ± 2.70	< 0.008
Hepatocellular carcinoma SMMC7721	16.69 ± 0.33	17.40 ± 0.51	< 0.008
Breast cancer MCF-7	16.78 ± 0.32	14.96 ± 0.45	< 0.008
Colon cancer SW480	13.58 ± 0.71	14.34 ± 1.15	< 0.008

Data expressed as mean ± SD (n = 3)

## Experimental

### General Experimental Procedures

Optical rotations were measured with a P-1020 polarimeter (JASCO, Tokyo, Japan). IR spectra were measured on Bio-Rad FTS-135 series spectrometer or NICOLLET iS10 with OMNIC 9.8.372 software. UV spectra were recorded on a Shimadzu UV2401A ultra-violet-visible spectrophotometer. HRESIMS spectra were run on an API QSTAR Pular-1 spectrometer. NMR spectra were measured in pyridine-*d*_5_ and recorded on a Bruker DRX-600 spectrometer, using TMS as an internal standard. Chemical shifts were reported in units of *δ* (ppm) and coupling constants (*J*) were expressed in Hz. Column chromatography (CC) were carried out over macro-porous resin D101 (Cangzhou Baoeng Co. Ltd., China), silica gel (200–300 mesh, Qingdao Haiyang Chemical Co. Ltd., China), RP-C18 gel (40–60/μm, Merck, Darmstadt, Germany). A Hanbon series (Hanbon Sci & Tech) were used for semi-preparative HPLC with a Capcell Pak MGII C18 column (5 μm, 250 mm × 10 mm).

### Plant Material

The rot roots of *P. notoginseng* (Burk.) F. H. Chen were collected in Wenshan County, Yunnan Province, People’s Republic of China, in February 2017, and identified by Prof. C. R. Yang from Kunming Institute of Botany (KIB), Chinese Academy of Sciences (CAS). A voucher specimen (KIB-Z-2017001) was deposited in the State Key Laboratory of Phytochemistry and Plant Resource in West China of KIB, CAS.

### Extraction and Isolation

The fresh rot roots of *P. notoginseng* (70 kg) were cut from the healthy part, air-dried under room temperature, and then extracted with CH_3_OH three times. After removal of the organic solvent, the methanol extract (4.0 kg) was subjected to macro-porous resin D101 CC (250 × 30 cm) eluting with H_2_O to remove most of the saccharides and then washing with MeOH to give a crude saponins fraction (2.7 kg), which was further fractionated on a silica gel CC (250 × 30 cm), eluting with CHCl_3_–CH_3_OH (7:3), to afford three fractions (Fr. A–C). TLC and HPLC analysis showed Fr. A (220.8 g) containing mainly notoginsenoside R_1_ and ginsenosides Rg_1_, Re, Rd and Rb_1_.

Fr. C (220.8 g) was applied to RP-18 CC, eluting with CH_3_OH–H_2_O (1:9 to 9:1), to yield six sub-fractions (Fr. C1–C6). Fr. C2 (1.2 g) was column chromatographed over silica gel CC, eluting with CHCl_3_–CH_3_OH (10:1) to give eight sub-fractions (Frs. C1.1–C1.8). Fr. C1.4 (37 mg) and Fr. C1.5 (21 mg) were separately purified with semi-preparative HPLC (CH_3_CN–H_2_O, 35:65) to yield **7** (2.9 mg) and **8** (10.0 mg), **4** (6.2 mg) and **6** (20 mg). Fr. C1.6 (21 mg) was purified with semi-preparative HPLC (CH_3_CN–H_2_O, 31:69) to yield **3** (4.2 mg). Fr. C3 (4 g) was subjected to a silica gel CC eluting with CHCl_3_–CH_3_OH (200:1 to 95:5), followed with semi-preparative HPLC (CH_3_CN–H_2_O, 35:65) to yield **1** (4.0 mg). Fr. C5 (1.1 g) was subjected to a silica gel CC eluting with CHCl_3_–CH_3_OH (200:1 to 10:1), followed with semi-preparative HPLC (CH_3_CN–H_2_O, 44:56) to yield **5** (2.8 mg) and **2** (2.5 mg).

#### 3*β*,6*α*,12*β*,20(*S*)-Tetrahydroxydammar-24-one-25-ene (**1**)

White amorphous powder; $[\alpha]_{\text{D}}^{25}$ − 24.83 (*c* 0.06, MeOH); IR (KBr) *ν*_max_: 3391, 2954, 2924, 2853, 1738, 1674, 1463, 1414, 1031; UV (MeOH) λ_max_ (log *ε*): 254 (0.10), 196 (0.35) nm; ^13^C and ^1^H NMR data: see Tables 1 and 2; HRESIMS (pos.): *m*/*z* 513.3551 [M+Na]^+^, (calcd. for C_30_H_50_O_5_Na, 513.3550).

#### 6*α*,20(*S*),24(*R*)-Trihydroxydammar-3,12-dione-25-ene (**2**)

White amorphous powder; $[\alpha]_{\text{D}}^{25}$ + 94.95 (*c* 0.08, MeOH); IR (ATR) *ν*_max_: 3478, 3452, 3405, 2956, 2916, 2880, 1690, 1111; UV (MeOH) λ_max_ (log *ε*): 195 (0.35) nm; ^13^C and ^1^H NMR data: see Tables 1 and 2; HRESIMS (pos.): *m*/*z* 511.3399 [M+Na]^+^, (calcd. for C_30_H_48_O_5_Na, 511.3399).

#### 3-Oxo-20(*S*)-ginsenoside-Rh_6_ (**3**)

White amorphous powder; $[\alpha]_{\text{D}}^{25}$ + 70.67 (*c* 0.12, MeOH); IR (ATR) *ν*_max_: 3356, 2956, 2934, 2880, 1692, 1425, 1380, 1076, 1035; UV (MeOH) λ_max_ (log *ε*): 195 (0.37) nm; ^13^C and ^1^H NMR data: see Tables 1 and 2; HRESIMS (pos.): *m*/*z* 691.4024 [M+Na]^+^, (calcd. for C_36_H_60_O_11_Na, 691.4033).

#### 3-Oxo-20(*S*)-ginsenoside Rh_1_ (**4**)

White amorphous powder; $[\alpha]_{\text{D}}^{25}$ + 61.42 (*c* 0.13, MeOH); IR (KBr) *ν*_max_: 3422, 2964, 2931, 2877, 1691, 1637, 1458, 1383, 1077, 1022; UV (MeOH) λ_max_ (log *ε*): 196 (0.52), 254 (0.07) nm; ^13^C and ^1^H NMR data: see Tables 1 and 2; HRESIMS (pos.): *m*/*z* 659.4126 [M+Na]^+^, (calcd. for C_36_H_60_O_9_Na, 659.4135).

### Acid Hydrolysis of Compounds **3** and **4**

Compounds **3** and **4** (each 2.0 mg) were hydrolyzed in 2 M HCl (3 mL) at 65 °C for 4 h, respectively. The reaction mixture was extracted with CH_2_Cl_2_, three times (3 × 3 mL). The aqueous layer was neutralized with 2 M NaOH and dried to produce a monosaccharide mixture. Next, l-cysteine methyl ester hydrochloride (1.0 mg) was added to a no aqueous pyridine solution of the sugar mixture (1.0 mL) and kept at 60 °C for 1 h. After this, trimethylsilylimidazole (1.0 mL) was added to the reaction mixture and kept at 60 °C for 30 min, followed the extraction with *n*-hexane (1 × 2 mL). The *n*-hexane layer was subjected to GC analysis, run on a Agilent Technologies HP5890 gas chromatograph, equipped with a HP-5 quartz capillary column (30 mm × 0.32 mm × 0.25 mm) and a H_2_ flame ionization detector with the following conditions: column temperature, 180–280 °C; programmed increase, 3 °C/min; carrier gas, N_2_ (1.5 mL/min); injector and detector temperature, 250 °C; injection volume, 2.0 μL; and split ratio 1/50. The configuration of the sugar moiety was determined by comparing the retention time with the derivatives of the authentic samples. The retention times of d-/l-glucose were 20.418/20.825 min, and the configuration of the respective sugar moiety from compounds **3** and **4** was determined as d-glucose.

### The Nitric Oxide Production in RAW264.7 Macrophages

As reported previously [31, 32], murine macrophage cell line RAW264.7 obtained from Cell Bank of Chinese Academy of Sciences (Beijing, People’s Republic of China), were seeded in 96-well cell culture plates (1.5 × 10^5^ cells/well) and treated with serial dilutions of the compounds with a maximum concentration of 50 μM in triplicate, followed by stimulation with 1 μg/mL LPS (Sigma, St. Louis, MO, USA) for 18 h. Nitric oxide production in the supernatant was assessed by Griess reagents (Reagent A & Reagent B, respectively, Sigma). The absorbance at 570 nm was measured with a microplate reader (Thermo, Waltham, MA, USA). NG-Methyl-l-arginine acetate salt (L-NMMA, Sigma), a well-known nitric oxide synthase (NOS) inhibitor, was used as a positive control. All the compounds were prepared as stock solutions in DMSO. The viability of RAW264.7 cells was evaluated by the MTS assay simultaneously to exclude the interference of the cytotoxicity of the test compounds. Concentration of a compound inhibiting 50% of cell growth (IC_50_) was calculated by the Reed and Muench method.

### Cytotoxic Assay

Five human cancer cell lines, myeloid leukemia HL-60, lung cancer A-549 cells, hepatocellular carcinoma SMMC7721, breast cancer MCF-7, and colon cancer SW480, were used in the cytotoxic assay. All the cells were cultured in RPMI-1640 or DMEM medium (Hyclone, USA), supplemented with 10% fetal bovine serum (Hyclone, USA). The cytotoxicity assay was performed according to the MTS [3-(4,5-dimethylthiazol-2-yl)-5(3-carboxymethoxy-phenyl)-2-(4-sulfopheny)-2H-tetrazolium] method in 96-well microplates [33]. Briefly, adherent cells (100 μL) was seeded into each well of 96-well cell culture plates and allowed to adhere for 24 h before drug addition, while suspended cells were seeded just before drug addition, each tumor cell line was exposed to the test compound dissolved in DMSO in triplicates for 48 h at 37 °C, with DDP and Taxol (Sigma, USA) as positive controls. After the incubation, 20 μL MTS and 100 μL medium were added to each well after removal of 100 μL medium, and the incubation continued for 4 h at 37 °C. The optical density was measured at 492 nm using a Multiskan FC plate reader (Thermo Scientific, USA). Concentration of a compound inhibiting 50% of cell growth (IC_50_) was calculated by the Reed and Muench method.

## Conclusion

In summary, eight PPT-type triterpenes (**1**–**2**, **5**, **8**) and saponins (**3**–**4**, **6**–**7**) were isolated from the rot roots of *P. notoginseng* for the first time. Among them, **1**–**4** are new compounds. Their structures were determined on the basis of the extensive spectroscopic analyses and acidic hydrolysis. Compound **1** with a novel terminal ketene at C-24 to C-26 showed significant cytotoxic and anti-inflammatory activities.

## Electronic supplementary material

Below is the link to the electronic supplementary material.

Supplementary material 1D and 2D NMR, HRESIMS, IR, UV, OR, and CD spectra of compounds **1-4**, and GC analysis of compounds **3**, **4** and d-/l-glucose of the corresponding trimethylsilylated l-cysteine adducts are available as Supporting Information (SI). (DOCX 5775 kb)
