# Supplementary material for: Anti-inflammatory and Cytotoxic Triterpenes from the Rot Roots of Panax notoginseng
Source: Nat Prod Bioprospect. 2019 May 23;9(4):287–95. doi: 10.1007/s13659-019-0211-4 (PMC6646631; doi:10.1007/s13659-019-0211-4)
Supplement: Supplementary file 1 — Supplementary material 1D and 2D NMR, HRESIMS, IR, UV, OR, and CD spectra of compounds 1-4, and GC analysis of compounds 3, 4 and d-/l-glucose of the corresponding trimethylsilylated l-cysteine adducts are available as Supporting Information (SI). (DOCX 5775 kb) [file 13659_2019_211_MOESM1_ESM.docx]

**Supporting Information**

**Anti-inflammatory and cytotoxic triterpene from the rot roots of *Panax notoginseng***

Jia-Huan Shang^1,2^, Guo-Wei Xu^1,2^, Hong-Tao Zhu^1,3^, Dong Wang^1,3^, Chong-Ren Yang^1^, Ying-Jun Zhang^1,3,^*

*^1^State Key Laboratory of Phytochemistry and Plant Resources in West China, Kunming Institute of Botany, Chinese Academy of Sciences, Kunming 650201, PR China*

*^2^University of Chinese Academy of Sciences, Beijing 100049, PR China*

*^3^Yunnan Key Laboratory of Natural Medicinal Chemistry, Kunming Institute of Botany, Chinese Academy of Sciences, Kunming 650201, PR China*

** Tel: +86-871-6522-3235. E-mail:* [*zhangyj@mail.kib.ac.cn*](mailto:zhangyj@mail.kib.ac.cn) *(Ying-Jun Zhang)*

| **No.** | **Content** | **Page** |
| --- | --- | --- |
| 1 | Figure S1. ^1^H NMR spectrum of compound **1** | 1 |
| 2 | Figure S2. ^13^C NMR spectrum of compound **1** | 1 |
| 3 | Figure S3. HSQC spectrum of compound **1** | 2 |
| 4 | Figure S4. ^1^H-^1^H COSY spectrum of compound **1** | 2 |
| 5 | Figure S5. HMBC spectrum of compound **1** | 3 |
| 6 | Figure S6. ROESY spectrum of compound **1** | 3 |
| 7 | Figure S7. HRESIMS of compound **1** | 4 |
| 8 | Figure S8. OR spectrum of compound **1** | 4 |
| 9 | Figure S9. UV spectrum of compound **1** | 5 |
| 10 | Figure S10. IR spectrum of compound **1** | 5 |
| 11 | Figure S11. CD spectrum of compound **1** | 6 |
| 12 | Figure S12. ^1^H NMR spectrum of compound **2** | 6 |
| 13 | Figure S13. ^13^C NMR spectrum of compound **2** | 7 |
| 14 | Figure S14. HSQC spectrum of compound **2** | 7 |
| 15 | Figure S15. ^1^H-^1^H COSY spectrum of compound **2** | 8 |
| 16 | Figure S16. HMBC spectrum of compound **2** | 8 |
| 17 | Figure S17. ROESY spectrum of compound **2** | 9 |
| 18 | Figure S18. HRESIMS of compound **2** | 9 |
| 19 | Figure S19. OR spectrum of compound **2** | 10 |
| 20 | Figure S20. UV spectrum of compound **2** | 10 |
| 21 | Figure S21. IR spectrum of compound **2** | 11 |
| 22 | Figure S22. CD spectrum of compound **2** | 11 |
| 23 | Figure S23. ^1^H NMR spectrum of compound **3** | 12 |
| 24 | Figure S24. ^13^C NMR spectrum of compound **3** | 12 |
| 25 | Figure S25. HSQC spectrum of compound **3** | 13 |
| 26 | Figure S26. ^1^H-^1^H COSY spectrum of compound **3** | 13 |
| 27 | Figure S27. HMBC spectrum of compound **3** | 14 |
| 28 | Figure S28. ROESY spectrum of compound **3** | 14 |
| 29 | Figure S29. HRESIMS of compound **3** | 15 |
| 30 | Figure S30. OR spectrum of compound **3** | 15 |
| 31 | Figure S31. UV spectrum of compound **3** | 16 |
| 32 | Figure S32. IR spectrum of compound **3** | 16 |
| 33 | Figure S33. CD spectrum of compound **3** | 17 |
| 34 | Figure S34. ^1^H NMR spectrum of compound **4** | 17 |
| 35 | Figure S35. ^13^C NMR spectrum of compound **4** | 18 |
| 36 | Figure S36. HSQC spectrum of compound **4** | 18 |
| 37 | Figure S37. ^1^H-^1^H COSY spectrum of compound **4** | 19 |
| 38 | Figure S38. HMBC spectrum of compound **4** | 19 |
| 39 | Figure S39. ROESY spectrum of compound **4** | 20 |
| 40 | Figure S40. HRESIMS of compound **4** | 20 |
| 41 | Figure S41. OR spectrum of compound **4** | 21 |
| 42 | Figure S42. UV spectrum of compound **4** | 21 |
| 43 | Figure S43. IR spectrum of compound **4** | 22 |
| 44 | Figure S44. CD spectrum of compound **4** | 22 |
| 45 | Figure S45. GC analysis of the sugar in **3** | 23 |
| 46 | Figure S46. GC analysis of the sugar in **4** | 23 |
| 47 | Figure S47. GC analysis of D-glucose | 24 |
| 48 | Figure S48. GC analysis of L-glucose | 24 |
| 49 | Figure S49. HPLC analysis of compounds **2** and **5** | 25 |


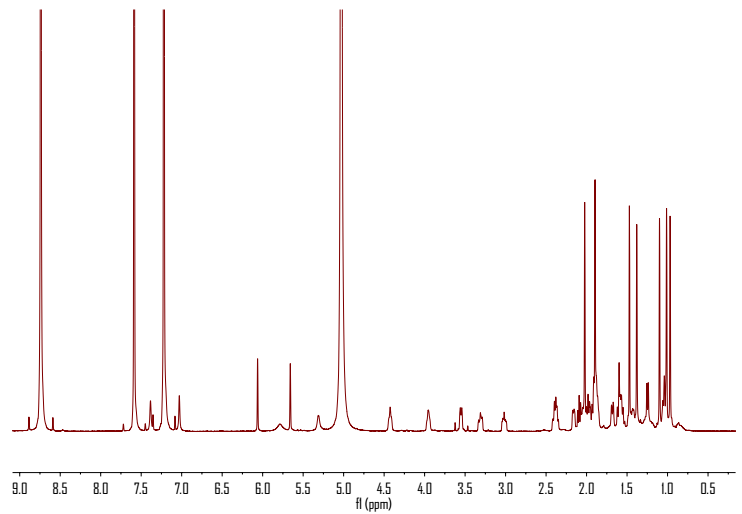
 **Figure S1.** ^1^H NMR spectrum of compound **1**


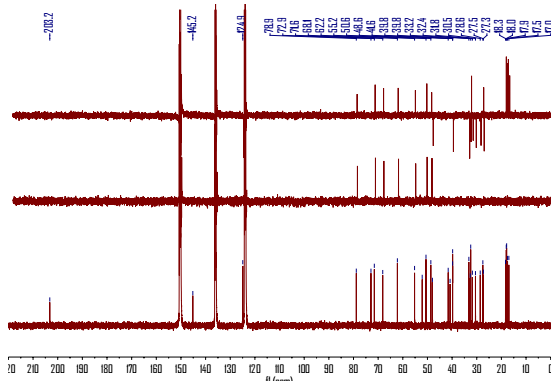
**Figure S2.** ^13^C NMR spectrum of compound **1**


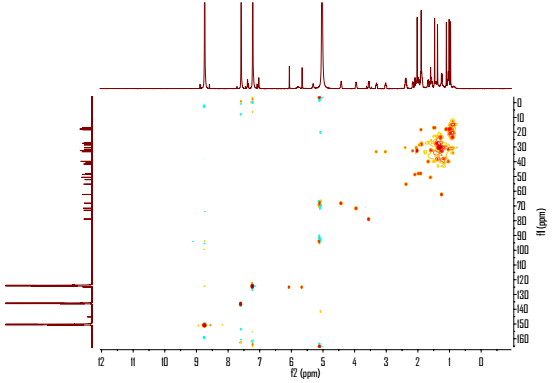
**Figure S3.** HSQC spectrum of compound **1**


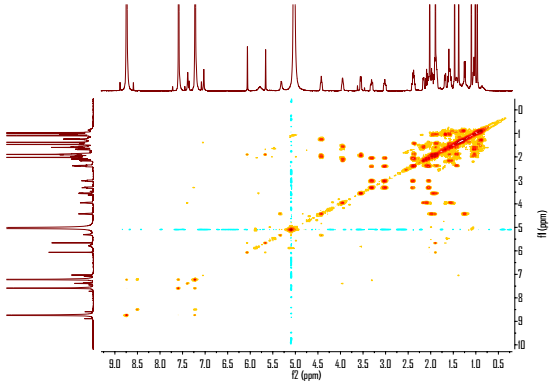


**Figure S4.** ^1^H-^1^H COSY spectrum of compound **1**


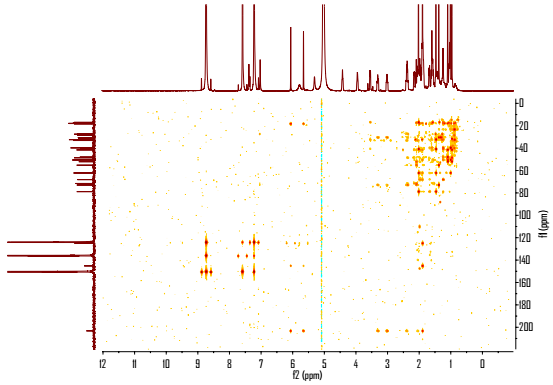


**Figure S5.** HMBC spectrum of compound **1**


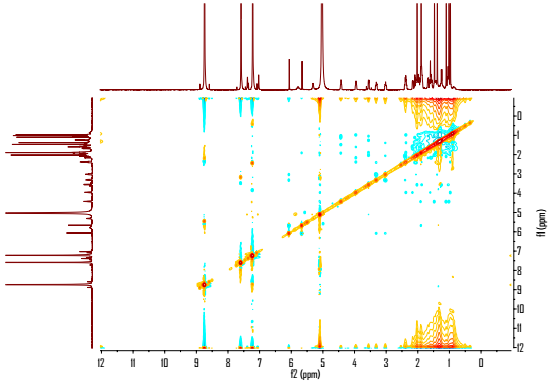


**Figure S6.** ROESY spectrum of compound **1**


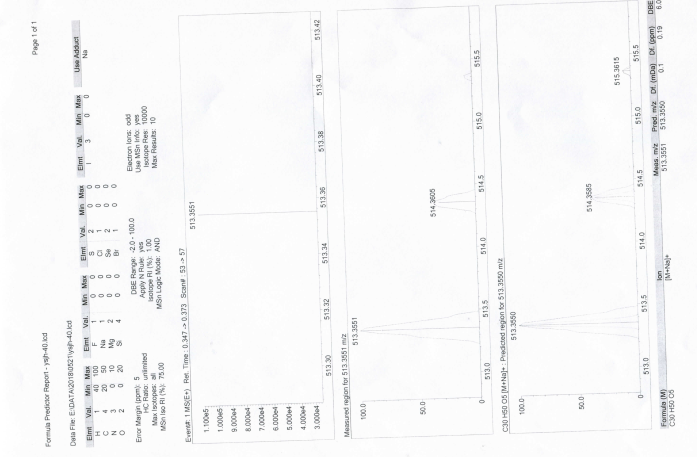


**Figure S7.** HRESIMS of compound **1**
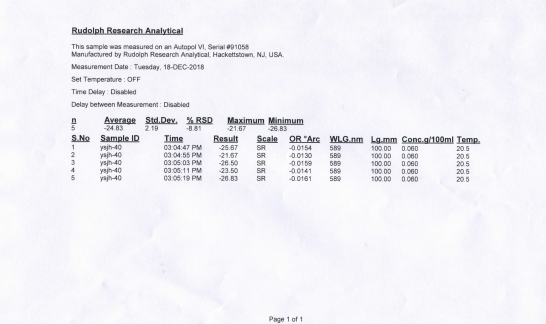


**Figure S8.** OR spectrum of compound **1**


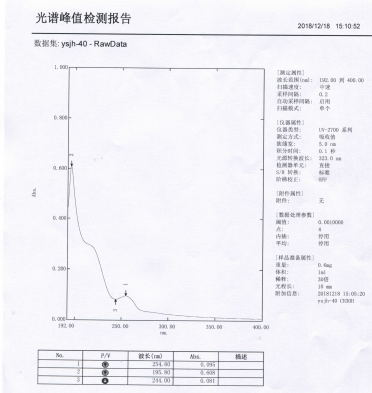


**Figure S9.** UV spectrum of compound **1**


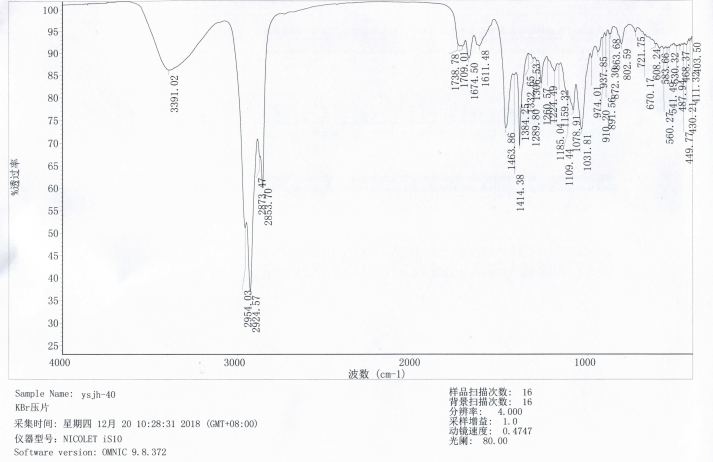


**Figure S10.** IR spectrum of compound **1**
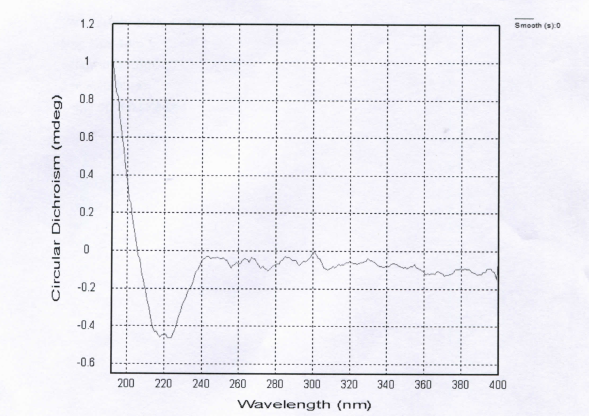


**Figure S11.** CD spectrum of compound **1**


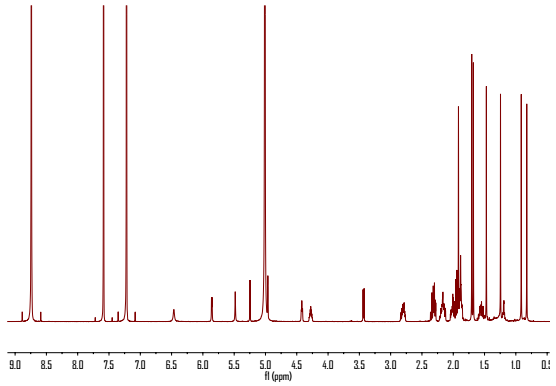


**Figure S12.**^1^H NMR spectrum of compound **2**


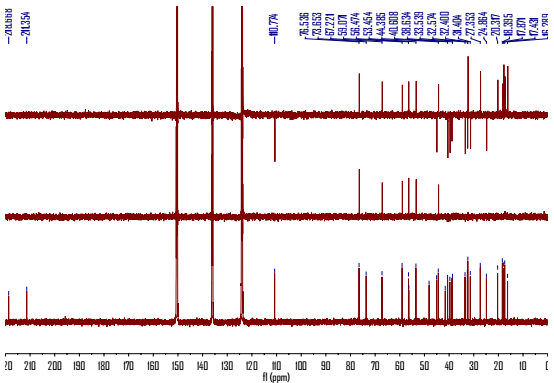
 **Figure S13.** ^13^C NMR spectrum of compound **2**


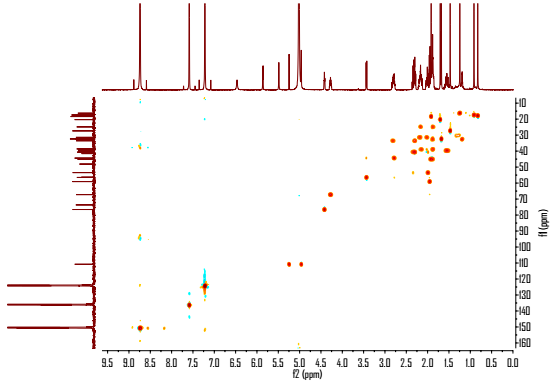
**Figure S14.** HSQC spectrum of compound **2**


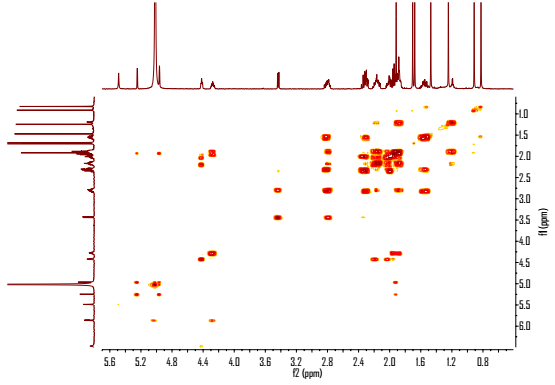
 **Figure S15.** ^1^H-^1^H COSY spectrum of compound **2**


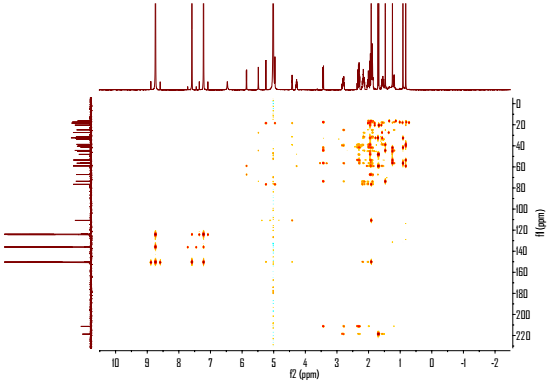


**Figure S16.** HMBC spectrum of compound **2**


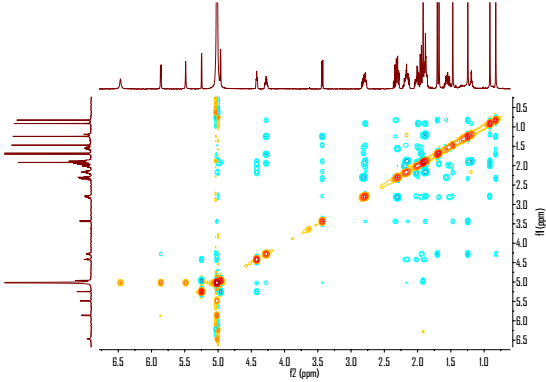
 **Figure S17.** ROESY spectrum of compound **2**


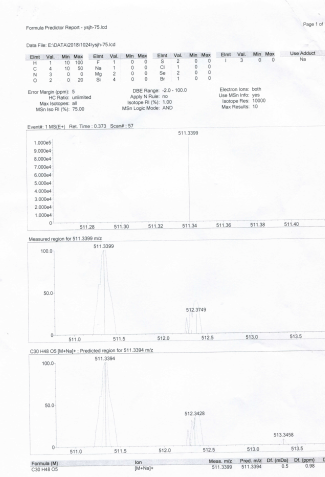


**Figure S18.** HRESIMS of compound **2**
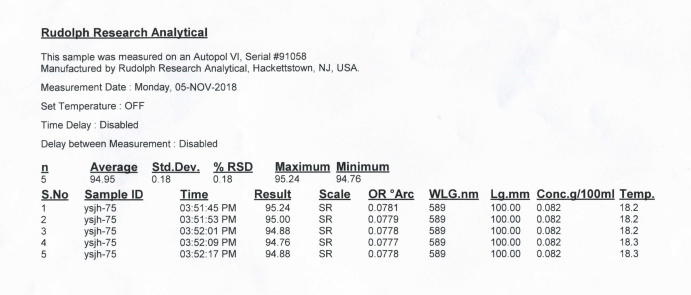


**Figure S19.** OR spectrum of compound **2**


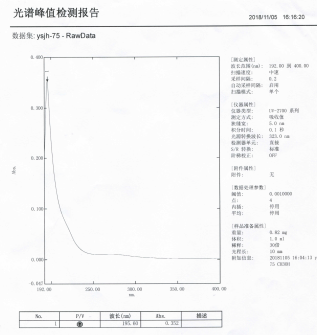


**Figure S20.** UV spectrum of compound **2**


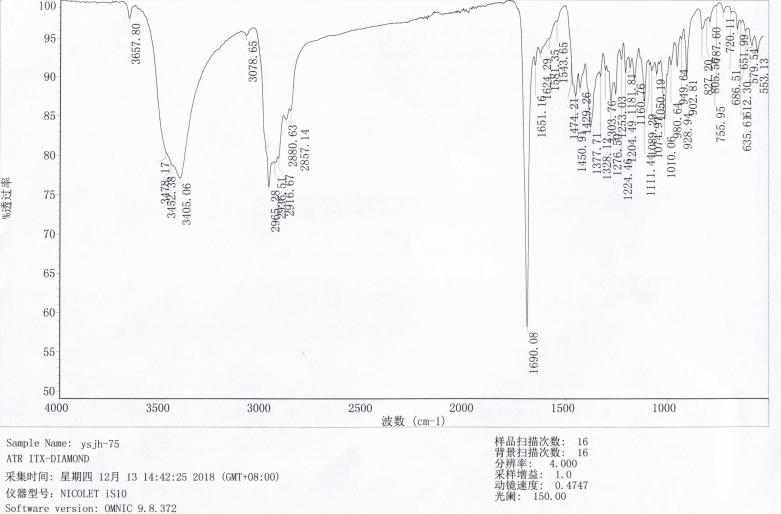


**Figure S21.** IR spectrum of compound **2**


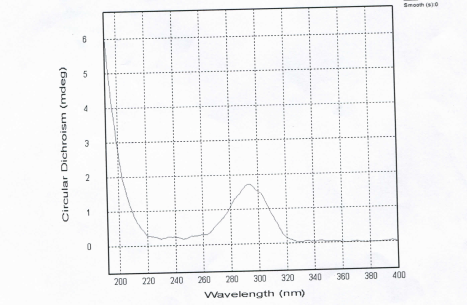


**Figure S22.** CD spectrum of compound **2**


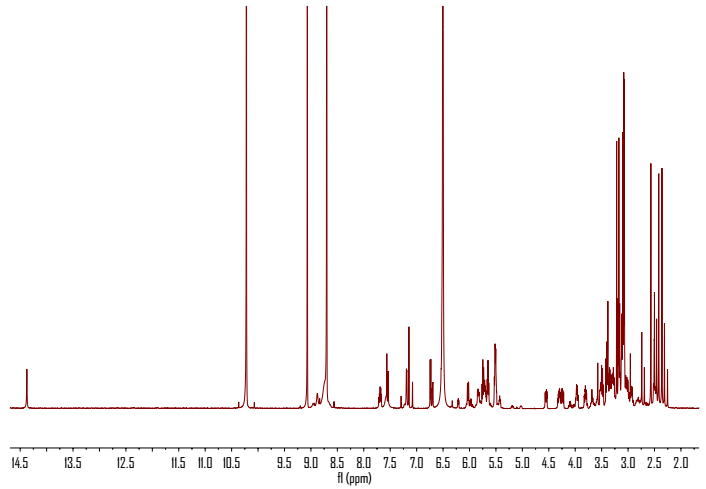


**Figure S23.** ^1^H NMR spectrum of compound **3**


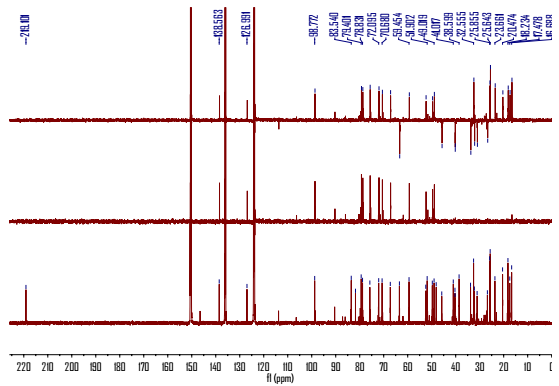


**Figure S24.** ^13^C NMR spectrum of compound **3**


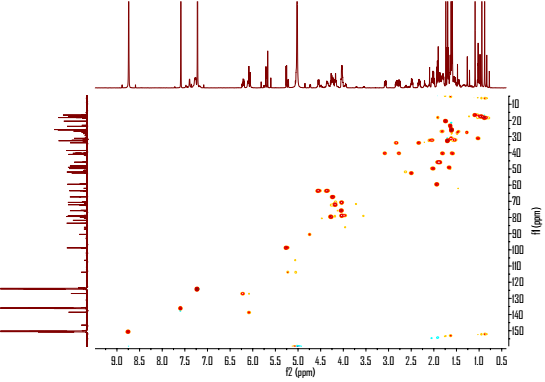


**Figure S25.** HSQC spectrum of compound **3**


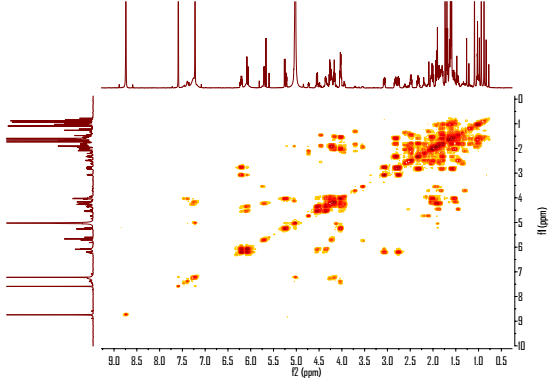


**Figure S26.** ^1^H-^1^H COSY spectrum of compound **3**


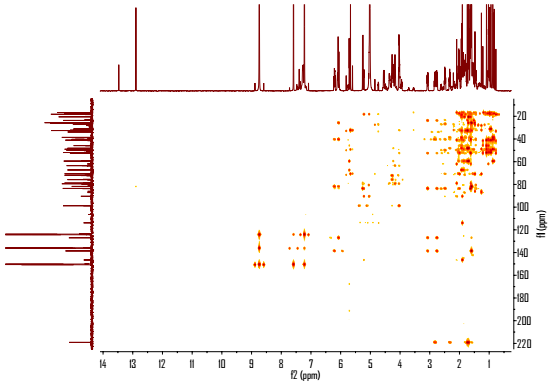


**Figure S27.** HMBC spectrum of compound **3**


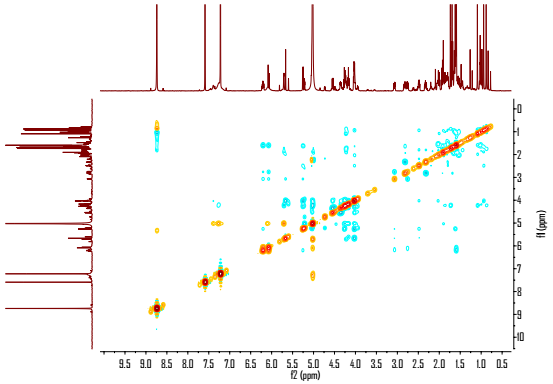


**Figure S28.** ROESY spectrum of compound **3**


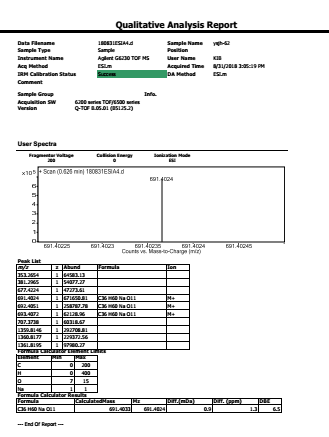


**Figure S29.** HRESIMS of compound **3**


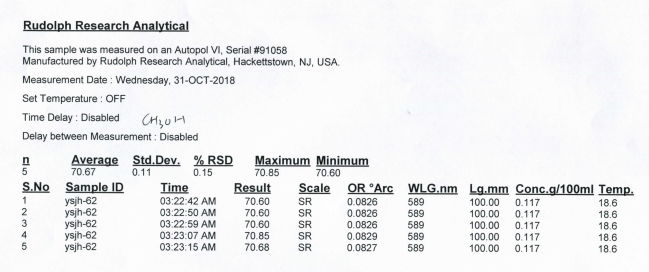


**Figure S30.** OR spectrum of compound **3**


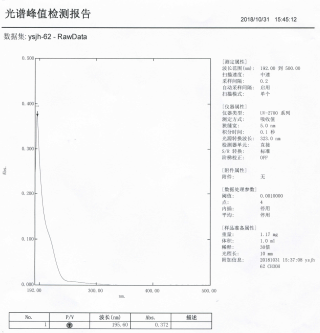


**Figure S31.** UV spectrum of compound **3**


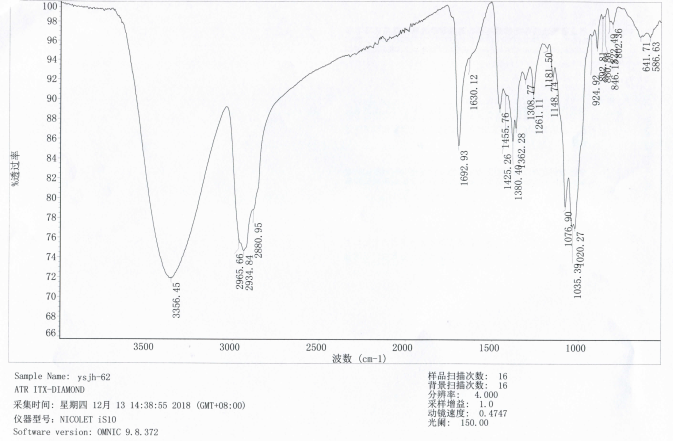


**Figure S32.** IR spectrum of compound **3**


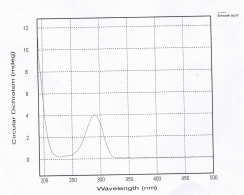


**Figure S33.** CD spectrum of compound **3**


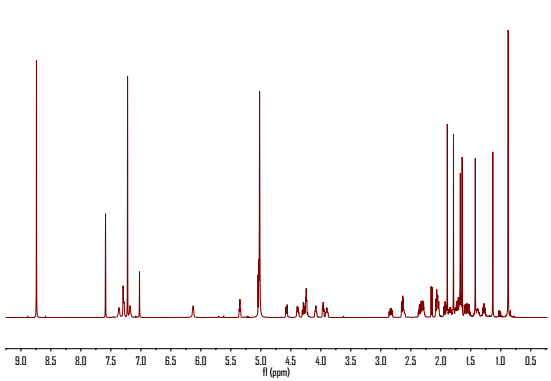


**Figure S34.** ^1^H NMR spectrum of compound **4**


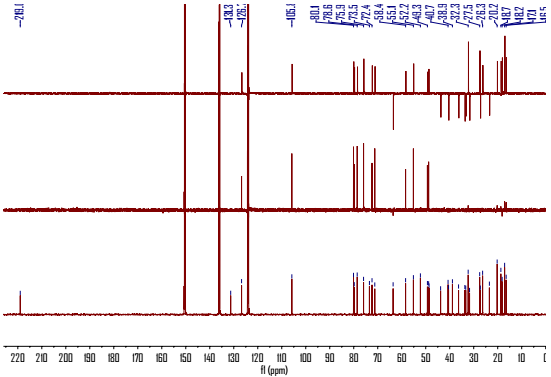


**Figure S35.** ^13^C NMR spectrum of compound **4**


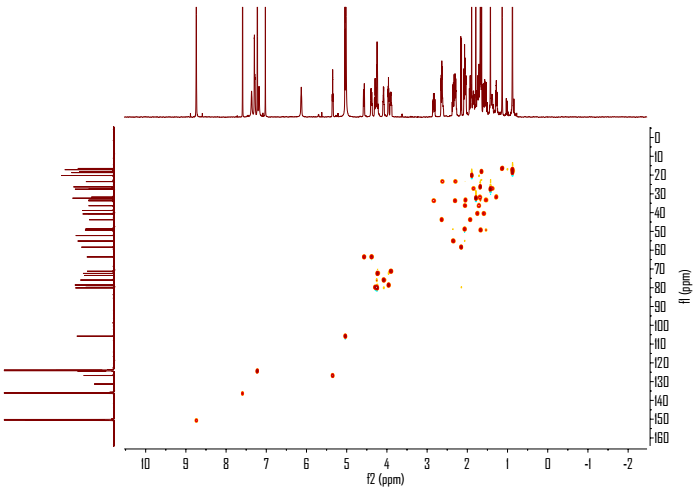


**Figure S36.** HSQC spectrum of compound **4**


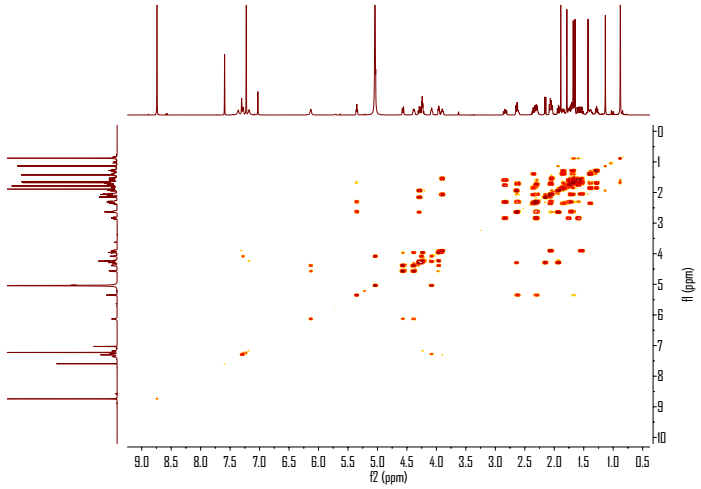


**Figure S37.** ^1^H-^1^H COSY spectrum of compound **4**


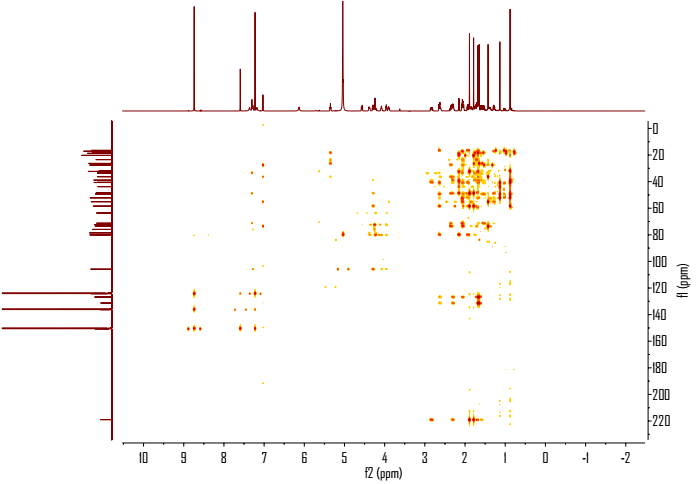


**Figure S38.** HMBC spectrum of compound **4**


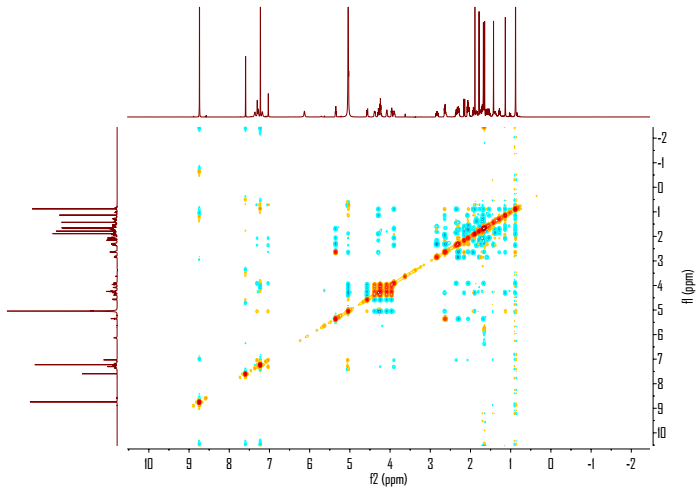


**Figure S39.** ROESY spectrum of compound **4**


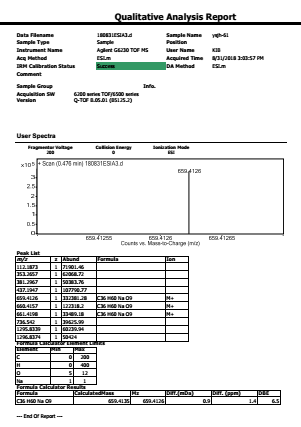


**Figure S40.** HRESIMS of compound **4**


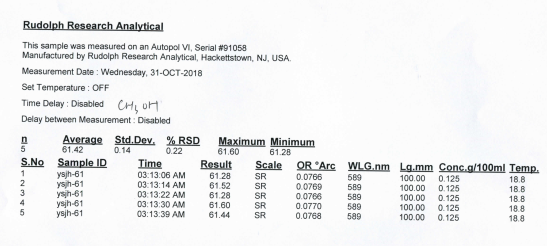


**Figure S41.** OR spectrum of compound **4**


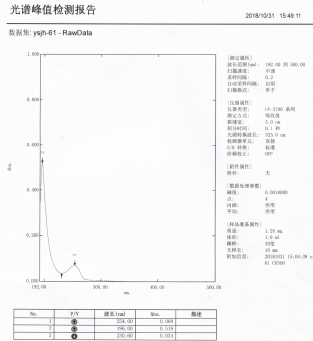


**Figure S42.** UV spectrum of compound **4**


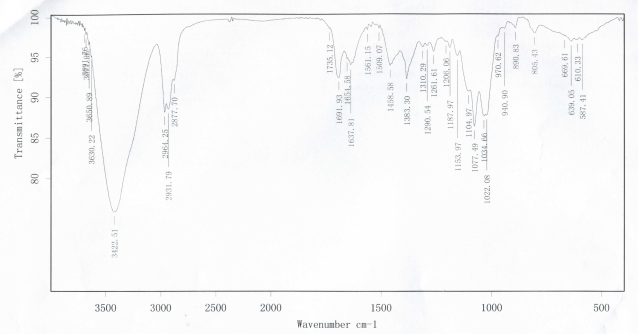


**Figure S43.** IR spectrum of compound **4**


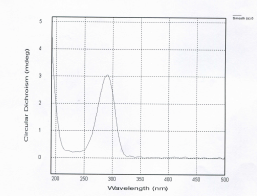


**Figure S44.** CD spectrum of compound **4**


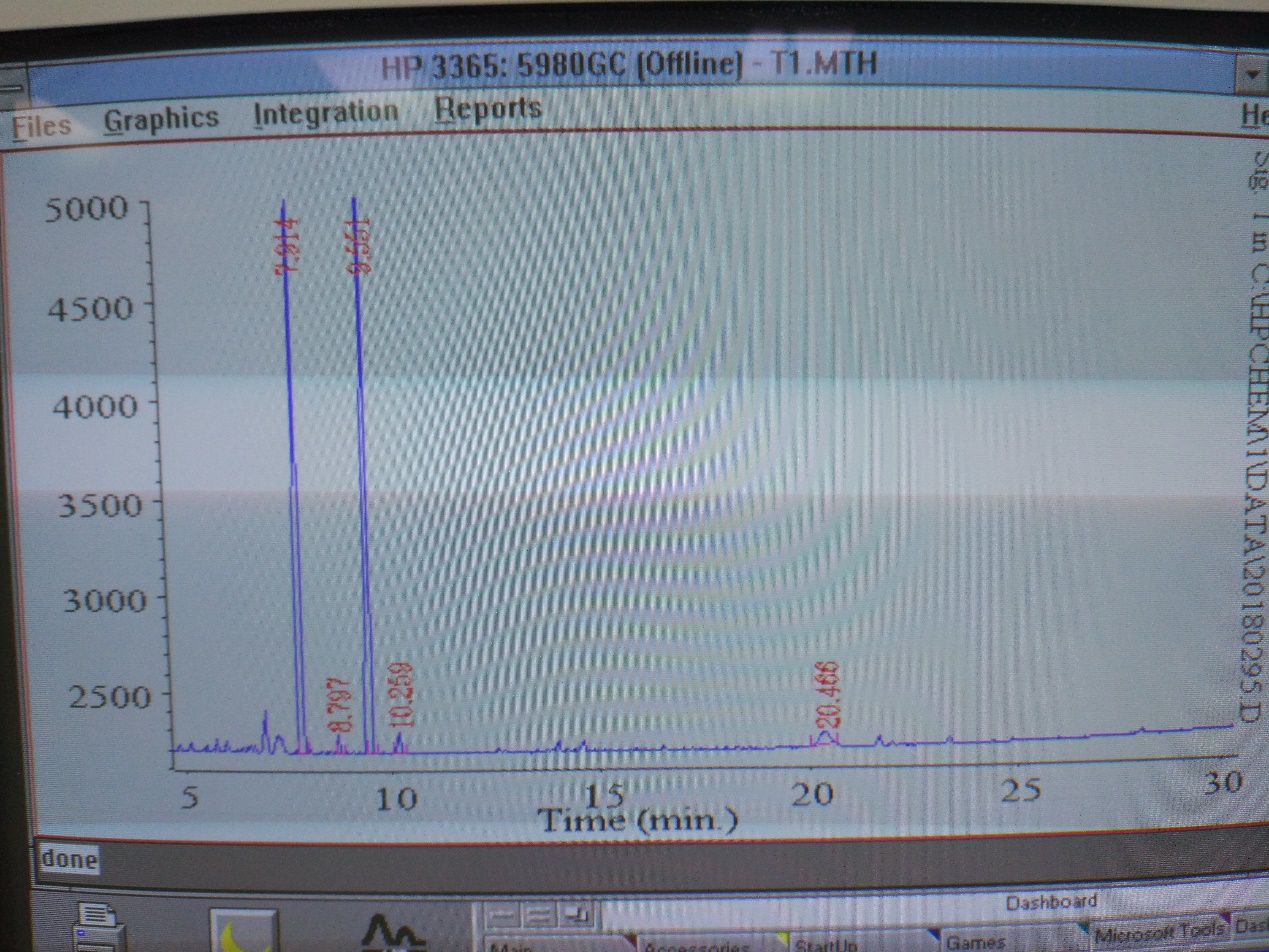


**Figure S45.** GC analysis of the sugar in **3**


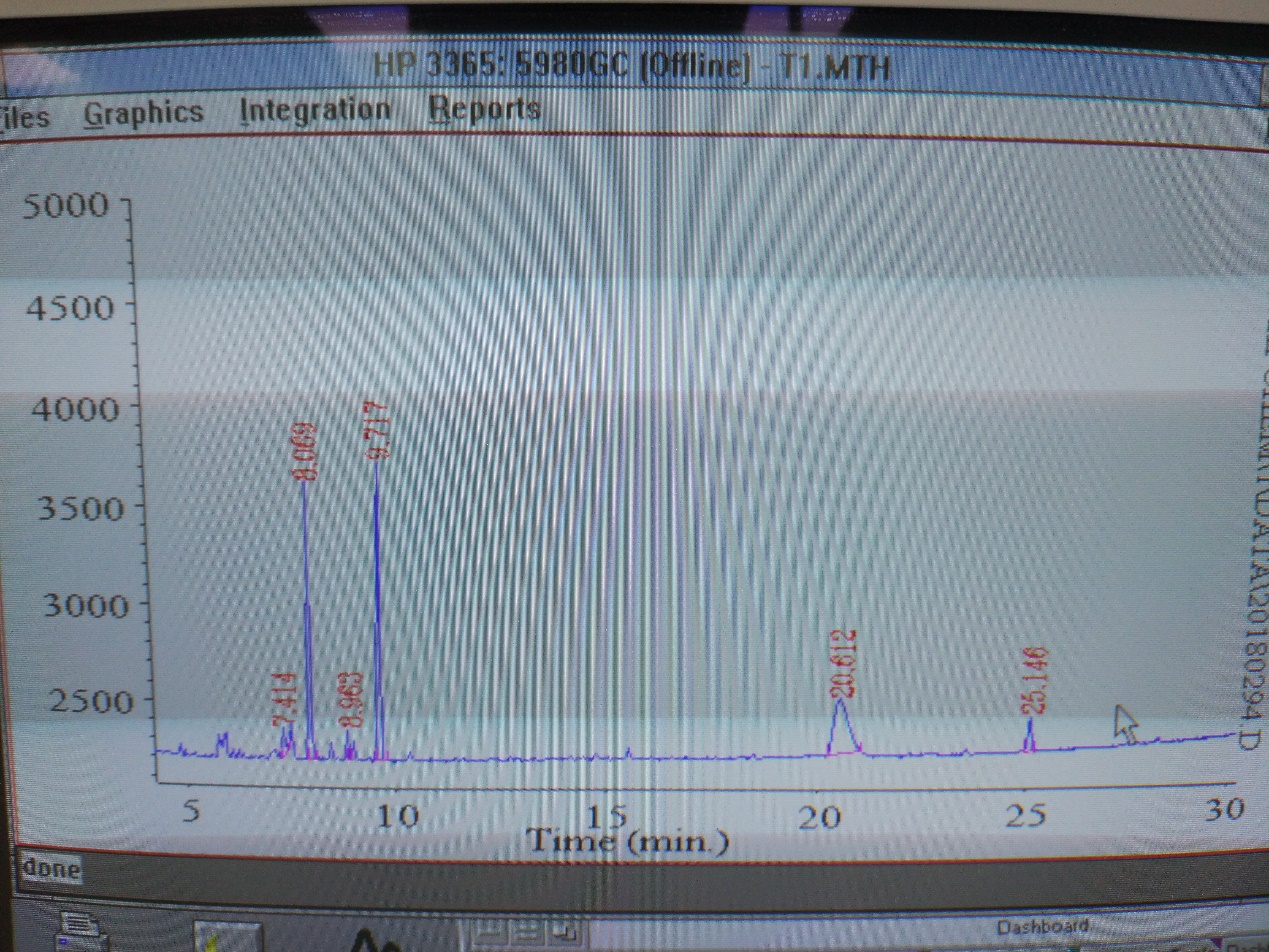


**Figure S46.** GC analysis of the sugar in **4**


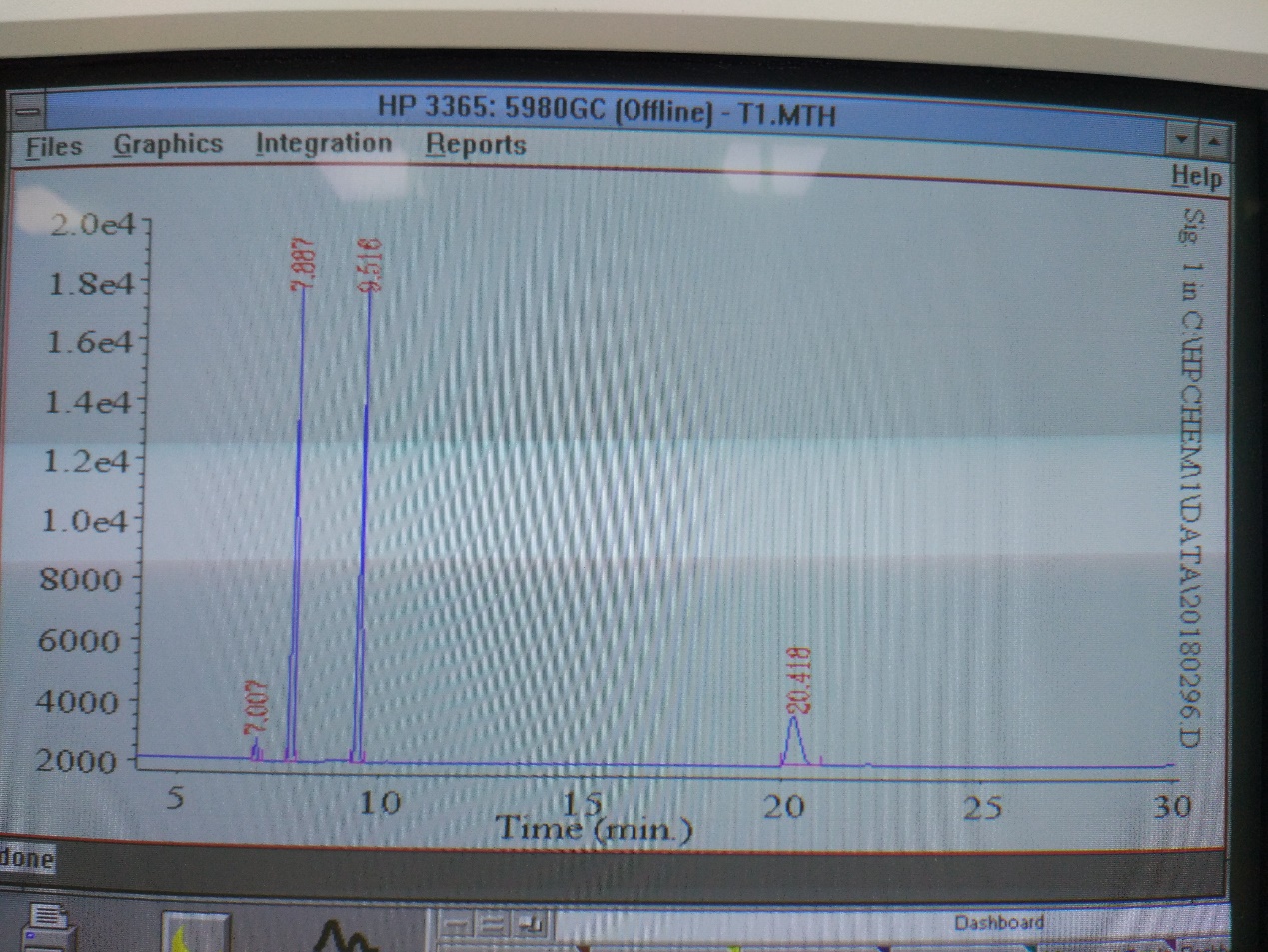


**Figure S47.** GC analysis of D-glucose


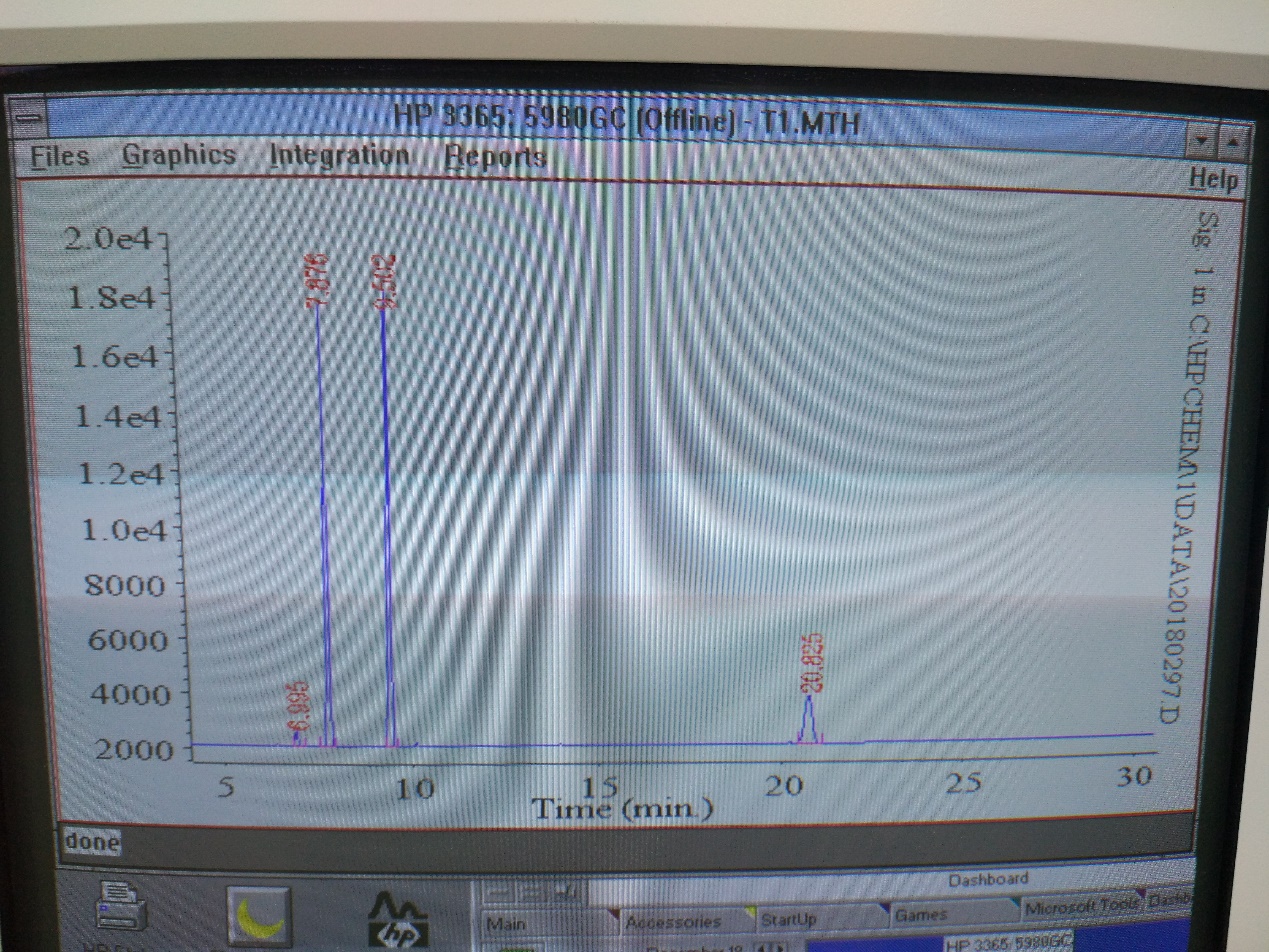


**Figure S48.** GC analysis of L-glucose

**Figure S49**. Pre-HPLC analysis of compounds **2** (31.587 min) and **5** (28.903 min)
